# Combined high-resolution assessment of quantitative perfusion and late enhancement. Towards accurate estimation of the ischaemic burden in patients with coronary artery disease

**DOI:** 10.1186/1532-429X-18-S1-Q15

**Published:** 2016-01-27

**Authors:** Adriana D Villa, Eva Sammut, Joy S Shome, Reza Razavi, Sven Plein, Amedeo Chiribiri

**Affiliations:** grid.13097.3c0000000123226764Cardiovascular Imaging, King's College London, London, United Kingdom

## Background

Cardiac magnetic resonance (CMR) allows the combined evaluation of myocardial perfusion status and viability in the same examination. Novel methods for high-resolution quantiative analysis of myocardial perfusion have rencently been described. High-resolution perfusion avoids spatial averaging and potentially enables a more accurate assessment of total inducible ischaemic burden. The simultaneous analysis of high-resolution late gadolinium enhancement images (LGE) allows excluding areas with overt ischaemic scar from quantitative perfusion analysis. This is potentially relevant to the estimation of the ischaemic burden, since areas of scar have can result in false-positive perfusion findings. The aim of this study was to test the feasibility of combined high-resolution assessment of perfusion and late enhancement, and to assess the potential of this novel approach to provide a more accurate estimation of the ischaemic burden in patients with coronary artery disease.

## Methods

20 patients with known CAD and previous myocardial infarct referred for stress perfusion CMR due to new onset of angina were included. All patients underwent rest and adenosine stress first-pass perfusion at 3T (Philips Achieva) using a high-resolution kt turbo field echo sequence and a dual bolus approach (0.075 mmol of Gd/kg of body weight; Gadovist). LGE images were acquired 15 minutes after the rest injection of Gd. Quantiative perfusion analysis was performed using validated high-resolution deconvolution analysis. LGE analysis was performed using conventional semi-quantitative analysis (signal threshold of 5 standard deviations). Stress and rest myocardial blood flow (MBF), myocardial perfusion reserve (MPR) and total LV ischaemic burden (MPR threshold of 1.5) were calculated with an without accounting for the presence of LGE and results compared.

## Results

All patients had LGE (scarred area 12.5 ± 5.7%). Before considering LGE, average stress MBF, rest MBF and MPR were 2.39 ± 0.94, 0.94 ± 0.42 and 2.83 ± 2.24, respectively. In areas of viable myocardium (LGE-), stress MBF, rest MBF and MPR were 2.82 ± 0.69,1.01 ± 0.26 (p = 0.01) and 3.25 ± 0.58, respectively. In areas of scar (LGE+), stress MBF, rest MBF and MPR were 1.02 ± 0.57,0.99 ± 0.74(p = ns) and 1.04 ± 0.63 (p = 0.001 vs LGE-), respectively. The overall ischaemic burden (at a MPR threshold of 1.5) was 21.3 ± 12.5%. When excluding areas with LGE, the ischaemic burden was 13.4 ± 6.7%(p = 0.001).

## Conclusions

This study demonstrates the feasbility of combined high-resolution assessment of quantitative perfusion and late enhancement. Areas of scar did not show any hyperhaemic flow during adenosine stress (MPR of approximately 1). When a threshold of 1.5 is used to define the ischaemic burden, these areas would incorrectly be ascribed to areas of inducible ischaemia if correction for the presence of LGE is not used. Combined high-resolution assessment of quantitative perfusion and late enhancement has the potential to provide more accurate assessment of myocardial perfusion status.

**Figure 1:**
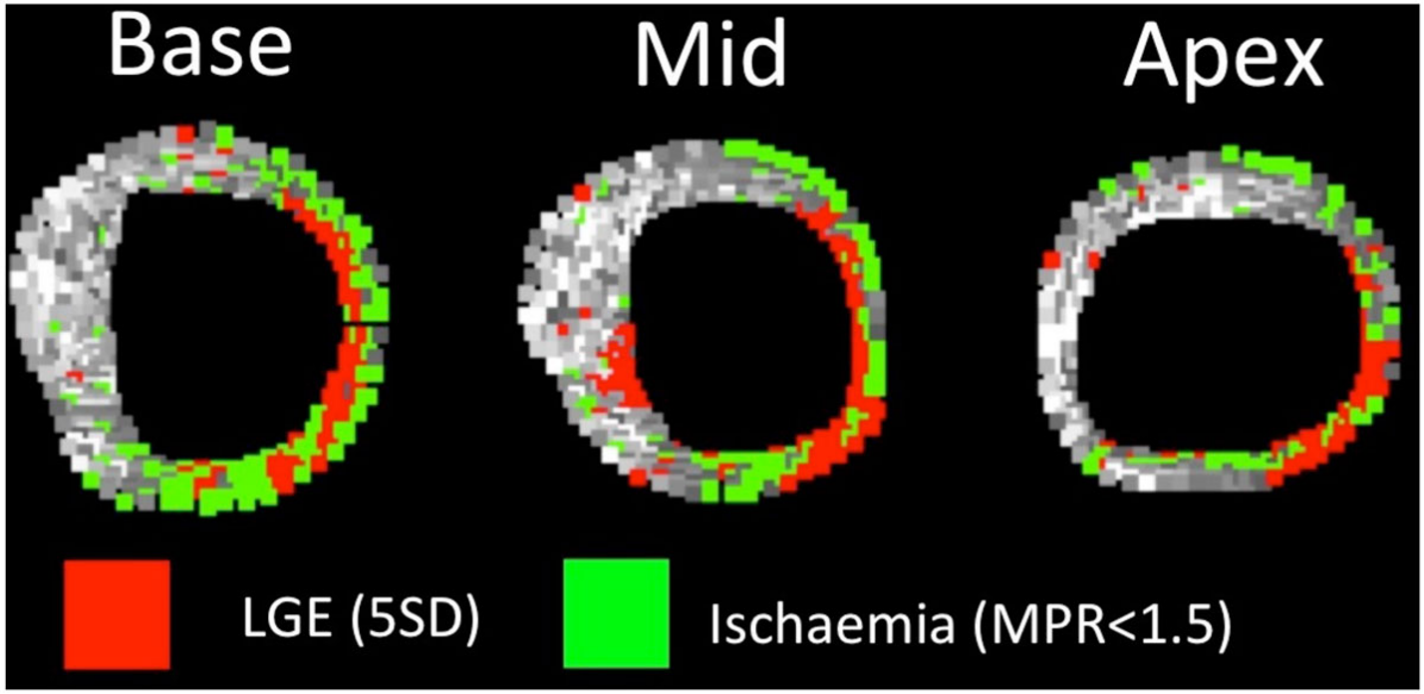
**High-resolution perfusion maps showing areas of scar in red and areas of ischaemia in green**.

